# Prevalence of overweight and obesity and their associations with blood pressure among children and adolescents in Shandong, China

**DOI:** 10.1186/1471-2458-14-1080

**Published:** 2014-10-17

**Authors:** Jing Dong, Xiao-Lei Guo, Zi-Long Lu, Xiao-Ning Cai, Hui-Cheng Wang, Ji-Yu Zhang, Liu-Xia Yan, Ai-Qiang Xu

**Affiliations:** Department of Non-communicable Disease Control and Prevention, Shandong Center for Disease Control and Prevention, 16992 Jingshi Road, Jinan, 250014 China; National Center for Chronic and Non-communicable Disease Control and Prevention, Chinese Center for Disease Control and Prevention, Beijing, 100050 China; Department of Tobacco Control, Chinese Center for Disease Control and Prevention, Beijing, 102206 China

**Keywords:** Prevalence, Overweight, Obesity, Adolescents, Blood pressure

## Abstract

**Background:**

Obesity and high blood pressure (BP) are public health problems all over the world. Some studies have reported a positive association between them in children and adolescents. The purpose of this study was to assess the prevalence of overweight and obesity and their associations with BP among school children and adolescents in Shandong, an important province in eastern China.

**Methods:**

In 2011, we conducted a cross-sectional population-representative survey in Shandong, China. A total of 4 898 children and adolescents aged 6–17 years were randomly selected from 140 counties/districts using a multistage random cluster sampling. Weight, height and BP were measured by a trained physician or pediatrician, and information about age, gender and place of residence was obtained using questionnaires. Obesity and high BP were defined according to age- and gender-specific Chinese reference data for children.

**Results:**

A total of 4 898 (100%) children and adolescents provided complete information. The prevalence of overweight, obesity and overweight plus obesity were 10.9%, 8.7% and 19.6%, respectively. Boys were more likely to be overweight or obese than girls (*P* < 0.05 for overweight; *P* < 0.001 for obesity). The prevalence of overweight plus obesity was highest among children aged 6–11 years (22.3%). BP and the prevalence of high BP increased with increasing body mass index (BMI). With age and sex adjusted, odds ratios (ORs) for high BP were [OR 2.2;95% CI 1.7–2.8) in overweight and [OR 3.6;95% CI 2.6–4.9] in obese children.

**Conclusion:**

The representative survey confirms high prevalence of overweight and obesity among children and adolescents in Shandong. Childhood obesity is a strong risk factor for high BP. Intervention programs should be implemented to combat the growing obesity epidemic.

## Background

Over the last few decades there has been a worldwide increase in childhood obesity [1, 2]. In the world, the prevalence of overweight and obesity among children and adolescents increased by 2.5% from 1990–2010. At the current rate of increase, the prevalence among the world’s children is expected to exceed 9% by 2020 [3]. Similar findings are being seen in China. In the past 30 years, China has gone through a rapid economic growth leading to expansion of urban areas and enhancement of living standards [4]. National data points to an increase in the prevalence of childhood obesity in recent years [5].

The etiology of childhood obesity is complex and influenced by genetic and environmental factors [6]. Childhood obesity increases the risk of adulthood obesity and is associated with other chronic diseases. National studies conducted in 2005 and 2010 indicated a positive association between blood pressure (BP) and obesity or body mass index (BMI) among Chinese children and adolescents [7, 8]. A similar study conducted by Zhang in Shandong in 2005 revealed the same findings [9, 10]. However, in the above mentioned study by Zhang, data was drawn from only three cities to represent the children and adolescents in Shandong, which was an important eastern littoral province with seventeen cities and a population of 96 million.

Therefore the study aimed to estimate the current prevalence of overweight and obesity and their associations with BP in a randomly selected representative sample among children and adolescents aged 6–17 years in Shandong.

## Methods

### Subjects

In 2011, we conducted a cross-sectional population-representative survey in Shandong, China. The target population was children and adolescents, aged 6–17 years, who lived in Shandong with their parents or guardians for at least 6 months. A total of 4 898 children and adolescents aged 6–17 years were randomly selected using a multistage random cluster sampling. First, a total of 140 counties/districts (in seventeen cities) in Shandong were stratified by geographic distribution (Eastern, Central-Southern, and North-Western), and by residence status (urban and rural). Second, using a proportional probability sampling (PPS) method, 1 primary school, 1 junior high school and 1 senior high school in urban areas and 2 primary schools, 2 junior high schools and 2 senior high schools in rural areas were selected from each geographic distribution. Then, one class was randomly selected from each grade in selected schools. Finally, all students aged 6–17 years in selected classes were eligible to participate in this study. A total of 27 schools, 81 classes, and 4 898 students were recruited between June 2011 and July 2011. All students voluntarily joined this study and written informed consent was obtained from the parents/guardians of the students. The study was approved by the Ethical Committee of the Shandong Center for Disease Control and Prevention.

### Anthropometric measurements

Anthropometric measurements including height and weight were conducted following the standardized procedures. Weight was measured with the subjects in light clothing (underwear or t-shirt) to the nearest 0.1 kg on a portable digital beam scale. Height was measured with the subjects barefooted and his/her back facing toward the wall to the nearest 0.1 cm using a tape measure and a square.

BMI was then calculated as weight in kilograms divided by the square of height in meters. The Working Group on Obesity in China (WGOC) definitions for childhood overweight and obesity (> = 85th and 95th percentile of BMI) were applied [11]. Subjects were classified by BMI as non-overweight (below the cut-off point for overweight), overweight (above the cut-off point for overweight) and obesity (above the cut-off point for obesity).

### BP measurements

Systolic blood pressure (SBP) and diastolic blood pressure (DBP) were measured after the subjects had rested for at least 5 minutes in a sitting position. BP was measured three times on the right arm using an electronic sphygmomanometer (HEM-7071, Omron Corporation, Japan) and appropriately sized cuff. Three readings were taken at one-minute intervals during which time the subjects were left alone. The arithmetic mean of three BP readings was calculated and used for analysis.

The age- and gender-specific BP cut-off points in Chinese children and adolescents were used to define relatively high BP status [12]. In this definition, high BP status was defined as SBP and/or DBP above the 95th percentile for age and gender.

### Statistical analyses

Descriptive statistics for height, weight, BMI, SBP, and DBP were calculated by gender and age group, and presented as the mean and standard deviation (SD). The prevalence of high BP, overweight, and obesity was presented as frequencies. To estimate the relationship between BP and BMI, SBP and DBP for age and BMI groups were also calculated. Weighted prevalence was estimated taking differential probabilities of selection and the complex sampling design into account. Comparisons of differences by gender and age groups were conducted using Pearson’s chi-square tests. Logistic regression was performed to calculate the odds ratios (ORs) and their 95% confidence intervals (CIs) of different BMI categories for high BP after adjusting for age and gender. All statistical tests were two-sided and considered statistically significant at a *p* value <0.05. Statistical analyses were performed using SPSS statistical package version 16 for windows (SPSS Inc., Chicago, IL, USA).

### Data quality control

A set of strategies was implemented to control data quality. All medical measurements were performed using the same type of apparatus by a trained physician or pediatrician. Health data collection sheets were checked every day to identify questionable information in time. Data was double entered into Epi-data 3.1 software, cleaning and archiving was conducted using established protocols.

## Results

Of the 4 898 children and adolescents enrolled into the study, completed usable data was available for 4 898 (Response rate 100.0%) subjects, comprising 2 534 boys and 2 364 girls, with a mean age of 11.7 ± 3.3 years. 35.3% of the subjects lived in urban areas.

**Figure 1:**
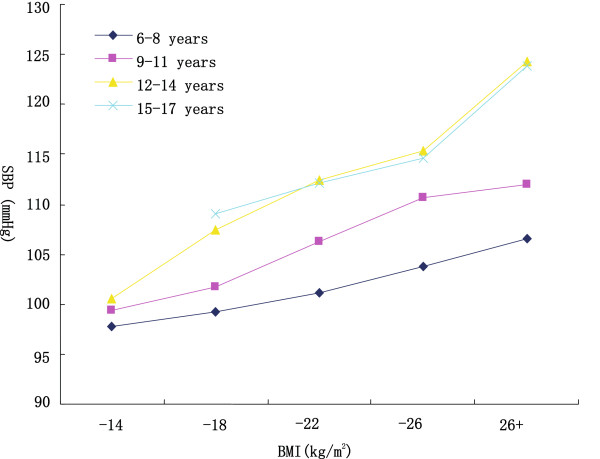
**Mean systolic blood pressure (SBP) in different body mass index (BMI) categories, age-specific.**

**Figure 2:**
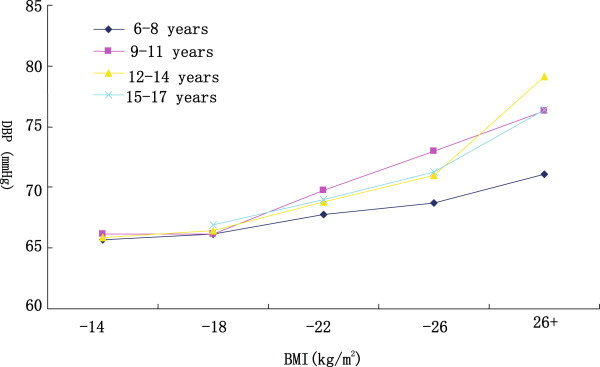
**Mean diastolic blood pressure (DBP) in different body mass index (BMI) categories, age-specific.**

Table 1 showed the descriptive characteristics of the subjects, as stratified by gender and age. Weight, height, BMI, SBP, and DBP increased with increasing age in both genders. Mean SBP increased by 17.5 mmHg and DBP increased by 4.4 mmHg in boys; SBP increased by 10.7 mmHg and DBP increased by 3.0 mmHg in girls from 6–8 years to 15–17 years age group, respectively. Mean BMI increased by 4.4 kg/m^2^ and 4.6 kg/m^2^ for boys and girls, respectively. Mean BMI of boys was higher than that of girls (t = 3.22, *p* < 0.05). As shown in Figures 1 and 2, both SBP and DBP increased in parallel with increasing BMI among all stratified age groups.

**Table 1:** **Descriptive characteristics of participants**

Age group(years)	Number	Height(cm)	Weight(kg)	BMI(kg/m ^2^)	SBP(mmHg)	DBP(mmHg)
Boys						
6–8	593	126.9 ± 7.4	27.2 ± 7.2	16.8 ± 3.7	99.8 ± 10.1	65.0 ± 8.8
9–11	599	140.5 ± 8.3	37.0 ± 10.8	18.5 ± 4.0	104.6 ± 10.9	67.6 ± 9.4
12–14	755	160.3 ± 9.8	51.3 ± 13.4	19.8 ± 3.9	113.3 ± 11.7	68.0 ± 8.7
15–17	587	171.6 ± 7.2	62.6 ± 13.1	21.2 ± 4.0	117.3 ± 11.6	69.4 ± 9.4
Girls						
6–8	481	125.9 ± 8.0	26.0 ± 6.3	16.2 ± 2.4	99.2 ± 10.7	66.7 ± 9.0
9–11	532	140.7 ± 8.4	34.6 ± 9.9	17.3 ± 3.8	103.0 ± 10.2	68.5 ± 8.8
12–14	703	156.8 ± 6.9	48.3 ± 11.5	19.6 ± 4.0	109.6 ± 10.9	69.6 ± 8.3
15–17	648	160.9 ± 6.6	53.6 ± 8.6	20.8 ± 5.0	109.9 ± 10.0	69.7 ± 8.7
All						
6–8	1074	126.5 ± 7.7	26.7 ± 6.9	16.5 ± 3.2	99.6 ± 10.4	65.7 ± 8.9
9–11	1131	140.6 ± 8.4	35.9 ± 10.5	17.9 ± 3.9	103.9 ± 10.6	68.0 ± 9.1
12–14	1458	158.6 ± 8.7	49.9 ± 12.6	19.7 ± 4.0	111.5 ± 11.5	68.8 ± 8.5
15–17	1235	166.0 ± 8.8	57.9 ± 11.9	21.0 ± 4.5	112.9 ± 10.0	69.7 ± 8.7

Note. Mean ± Standard Deviation; BMI, Body mass index; SBP, Systolic blood pressure; DBP, Diastolic blood pressure.

The prevalence of overweight and obesity among children and adolescents aged 6–17 years in Shandong were presented in Table 2. In 2011, the crude prevalence of overweight, obesity and overweight plus obesity were 10.9%, 8.7% and 19.6%, while the weighted prevalence were 11.0%, 7.7% and 18.7%, respectively. Boys were significantly more likely to be overweight or obese than girls (12.0% vs 9.8%, *P* < 0.05 for overweight; 10.9% vs 6.2%, *P* < 0.001 for obesity). There were some differences in age trend between prevalence of overweight and obesity. Across age groups, prevalence of overweight ranged from 10.3% to 11.9% (*P* >0.05); whereas prevalence of obesity was elevated with increasing age at first, reached to the highest percentage (12.0%) among children aged 9–11 years, then decreased to 5.3% among those aged 15–17 years (*P* < 0.001). A similar trend was observed when prevalence of overweight plus obesity was broken down by age. There was no significant difference in prevalence of overweight and obesity between urban and rural children.

**Table 2:** **Prevalence of overweight and obesity among children and adolescents aged 6–17 years in Shandong**

Variable	Boys (%)			Girls (%)			All (%)		
	Overweight	Obesity	Both	Overweight	Obesity	Botha	Overweight	Obesity	Both
Age group (years)									
6 ~ 8	12.1	11.1	23.2	9.6	11.2	20.8	11.0	11.2	22.2
9 ~ 11	11.5	16.2	27.7	8.8	7.3	16.1	10.3	12.0	22.3
12 ~ 14	13.6	8.7	22.3	10.0	5.3	15.3	11.9	7.1	19.0
15 ~ 17	10.2	8.2	18.4	10.5	2.6	13.1	10.4	5.3	15.7
Residence area									
Urban	12.9	12.0	24.9	11.9	6.1	18.0	12.4	9.1	21.5
Rural	11.5	10.3	21.8	8.6	6.3	14.9	10.1	8.4	18.5
Total	12.0	10.9	22.9	9.8	6.2	16.0	10.9	8.7	19.6
	12.1^a^	9.4 ^a^	21.5 ^a^	9.7 ^a^	5.8 ^a^	15.5 ^a^	11.0	7.7	18.7

Note. Both, Overweight + Obesity.

^a^Weighted prevalence was calculated taking into account differential probabilities of selection and the complex sampling design.

Table 3 showed prevalence of high BP among different BMI categories. The prevalence of high BP in non-overweight, overweight and obesity groups were 11.4%, 24.9% and 38.7%, respectively. Overweight and obese children had higher BP and showed a significantly higher prevalence of high BP than non-overweight group (*P* < 0.001). The adjusted ORs for high BP increased with increasing obese status classified by BMI categories.

**Table 3:** **Prevalence of high BP among different BMI Categories**

Gender	BMI Categories	SBP(mm Hg)	DBP(mm Hg)	High BP(%)	OR(95% CI)
Boys	Non-overweight	107.7 ± 12.1	66.3 ± 8.3	10.7	1.0
	Overweight	112.3 ± 13.4	70.0 ± 9.7	25.0	2.0(1.4,2.8)
	Obesity	114.9 ± 16.2	73.8 ± 11.2	38.6	2.7(1.8,4.1)
	Total	109.0 ± 13.0	67.5 ± 9.2	15.4	
Girls	Non-overweight	105.1 ± 11.0	68.0 ± 8.4	12.1	1.0
	Overweight	108.7 ± 11.6	71.0 ± 8.8	24.7	2.4(1.7,3.3)
	Obesity	110.7 ± 12.9	74.4 ± 10.2	38.8	4.2(2.9,6.1)
	Total	105.8 ± 11.3	68.8 ± 8.7	15.0	
Total	Non-overweight	106.4 ± 11.6	67.2 ± 8.4	11.4	1.0
	Overweight	110.7 ± 12.8	70.4 ± 9.4	24.9	2.2(1.7,2.8)
	Obesity	113.4 ± 15.2	74.0 ± 10.9	38.7	3.6(2.6,4.9)
	Total	107.5 ± 12.3	68.1 ± 9.0	15.2	

Note. OR, Odds Ratio; CIs, confidence intervals.

## Discussion

In this cross-sectional study, we examined the prevalence of overweight and obesity and their relationship with BP in a randomly selected representative sample among children and adolescents aged 6–17 years in Shandong. Our study showed that a considerable proportion (19.6%) of children and adolescents was overweight or obese. Compared with non-overweight children, those who were overweight or obese showed a significantly higher prevalence of high BP.

At present, childhood obesity has become a serious public health problem in the world. In the United States, up to 18% of children and adolescents are obese [13]. Prevalence of overweight plus obesity has been reported to be 20–30% in some European countries, including Spain, Italy, and Greece [14–16]. Similar findings are being seen in developing countries as well. 15.7% of adolescents in Brazil are reported to have excess weight [17]. In China, the prevalence of obesity increased from 0.13% and 0.12% in 1985, to 1.4% and 0.9% in 1995, and to 5.1% and 2.7% in 2005, for boys and girls among children and adolescents aged 7–18 years [18].

The prevalence of overweight plus obesity obtained in our study exceeded the results of the China Health and Nutrition Survey (CHNSs) in 2009(18.0%) [19], but was lower than that of studies in large cities in recent years, such as Shanghai(24.8%) and Tianjin(28.2%) [20, 21]. It may be possible that only 1108 children aged 7–18 years from 9 provinces participated in the CHNSs, which mean a small sample size. Compared to the results from Shanghai and Tianjin, the differences may be explained by different economic status. The prevalence of overweight plus obesity was the highest (one in five) among children aged 6–11 years. The finding was in line with other studies [22]. The special patterns of growth and maturation during adolescence may help explain the phenomena. A rapid increase in percentage of body fat and peak bone accretion may be observed among children aged 6–11 years. In our study, the prevalence of overweight and obesity was higher in boys than in girls. Gender-specific disparities in childhood obesity had been documented worldwide [23, 24]. Previous studies showed that differences in sedentary behaviors, genetics, and socio-cultural and economic factors may account for some of these disparities [25]. In China, traditional ideas of preferring boys over girls leading to disparities in food availability may be one of the unique reasons. Studies done in developed countries showed that rural children had a higher risk of overweight and obesity than urban children, whereas developing countries showed a reverse association [26, 27]. Our study indicated that children from rural areas were similar to their peers from urban areas in obesity epidemic. One of the main reasons maybe the decrease in urban–rural disparities. In addition, urban schools and parents may pay more attention on children obesity than rural ones.

Previous studies led to conflicting results as to whether there was an association between obesity and high BP in children. A study conducted in Changsha indicated that the risk ratio (RR) of hypertension were significantly higher in overweight (RR: 2.8, 95% confidence interval (CI): 2.6–3.2) and obese (RR: 8.7, 95% CI: 8.1–9.5) adolescents adjusted for age, sex, and height [28]. Another study of 8 568 students aged 7–18 years conducted in Shandong in 2005 reported that prevalence of relatively high BP increased with BMI percentiles and this trend was especially obvious in the upper percentiles of BMI [10]. However, a study conducted in Anhui showed no relationship between measures of adiposity and DBP among rural children aged 12–18 years [29]. The variations in adiposity measures, dietary intake, socioeconomic status, and physical activity may lead to these different results [28].

The study had several strengths. First, the database came from a large representative sample of children and adolescents in Shandong. We were able to analyze data separately in gender- and age-specific subgroups. Second, a set of strategies was implemented to ensure the high quality of the data, such as objectively measured BMI. However, several limitations should be considered. First, since the current study was cross-sectional, it was impossible to establish the causality relationships. Second, overweight, obesity and high BP were defined by Chinese criteria in the study. Therefore, results of the study might not be comparable with other findings.

## Conclusions

In conclusion, this study demonstrated that the prevalence of childhood obesity was high in Shandong, one of the populous provinces in China. BP and prevalence of high BP were significantly higher in overweight/obese children than in non-overweight ones, suggesting that overweight/obesity was a strong risk factor. Obesity has become a health threat to children and adolescents in Shandong, and as the urbanization increases, childhood obesity might show further increase. Thus, urgent effort should be made to combat the growing childhood obesity epidemic.
